# Periodontal inflammation and tryptophan-kynurenine metabolism in Parkinson’s disease

**DOI:** 10.1007/s00784-026-06853-4

**Published:** 2026-03-25

**Authors:** Melis Yilmaz, Aslihan Bakici, Rumeysa Parlat, İpek N. Akpinar, Rabia Karaaslan, Nur Balci, Hilal Toygar, Şivge Kurgan, Muhittin A. Serdar, Alpdoğan Kantarcı

**Affiliations:** 1 Department of Periodontology, Faculty of Dentistry, Medipol University, İstanbul, Turkey; 2 Institute of Health Sciences, Istanbul Medipol University, Istanbul, Turkey; 3 Department of Periodontology, Faculty of Dentistry, Ankara University, Ankara, Turkey; 4 Institute of Health Sciences, Ankara University, Ankara, Turkey; 5 Department of Medical Biochemistry, School of Medicine, Acibadem University, Ankara, Turkey; 6 School of Dental Medicine, Department of Developmental and SurgicalSciences, University of Minnesota, Minneapolis, MN USA; 7 School of Dental Medicine, Harvard University, Boston, MA USA

**Keywords:** Parkinson's disease, Periodontitis, Inflammation, Tryptophan metabolism, Kynurenine pathway

## Abstract

**Objective:**

The kynurenine pathway(KP) of tryptophan catabolism is a major regulator of the immune response. Metabolites of this pathway may have protective or degenerative effects on the nervous system. Based on our recent studies, we tested the hypothesis that KP metabolites play a role in the pathogenesis of the link between Parkinson’s disease (PAD) and Periodontitis (PD).

**Materials and methods:**

Saliva and serum samples were collected from Stage III, Grade B periodontitis patients with PAD (Parkinson+periodontitis group, *n* = 20) and without PAD (periodontitis group, *n* = 24), and 24 periodontally and systemically healthy individuals (control group). Salivary and serum concentrations of tryptophan-kynurenine pathway metabolites, including tryptophan, kynurenine, kynurenine/tryptophan ratio, kynurenic acid, 3-hydroxykynurenine, picolinic acid, and quinolinic acid, were quantified using liquid chromatography–mass spectrometry. Periodontal status was assessed by recording plaque index, probing pocket depth, clinical attachment loss, and bleeding on probing according to standard clinical procedures.

**Results:**

Clinical parameters were significantly higher in the PD groups than in the control group (*p* < 0.001). The control group had the lowest BOP (3.54 ± 2.52), followed by the Parkinson+periodontitis (52.00 ± 14.91) and the periodontitis groups (70.46 ± 25.09). Salivary TRP, KYN, KYNA, PA, and QA levels were significantly higher in the Parkinson+periodontitis group than in the control. The salivary KYN/TRP ratio was significantly higher in the Parkinson+periodontitis group than in the other groups (*p* < 0.05). Serum TRP levels were significantly higher in the periodontitis group compared to the other groups. The serum KYN/TRP ratio was significantly higher in the Parkinson+ periodontitis group than in the control group (*p* < 0.05).

**Conclusions:**

The data suggest that the metabolic regulation of immune responses via the tryptophan-kynurenine pathway may play a pathogenetic role in the link between Parkinson’s disease and periodontitis.

**Clinical relevance:**

Altered tryptophan–kynurenine metabolism in patients with both Parkinson’s disease and periodontitis suggests a shared inflammatory pathway linking the two conditions.

Clinical Trials ID: NCT07272564.

## Introduction

Parkinson’s disease (PAD) is a progressive neurodegenerative disease characterized by the loss of dopaminergic neurons in the substantia nigra region of the brain [1]. Although various mechanisms are considered in the pathogenesis of Parkinson’s disease, studies on the contribution of neuroinflammation are increasing.

Tryptophan (TRP) is an essential amino acid that contributes to protein synthesis, serotonin and melatonin production, and maintenance of membrane protein integrity within the cell membrane [2]. More than 95% of tryptophan that is not used in protein synthesis is catabolized to kynurenine [3]. Tryptophan metabolism through the kynurenine pathway is primarily regulated by two enzymes, tryptophan 2,3-dioxygenase (TDO) and indoleamine 2,3-dioxygenase (IDO). While TDO is considered the principal determinant of serotonergic metabolism, IDO is widely recognized for its immunomodulatory role, including cytokine-mediated anti-inflammatory effects [4]. The essential role of the KP is the biogenesis of nicotinamide adenine dinucleotide (NAD), and it was ascribed to its role in immune regulation and neurological disorders [5]. Several neuroactive compounds are produced through the KP that can be either neurotoxic, neuroprotective, or immunomodulatory.

Up-regulation or dysregulation of the KP of tryptophan catabolism is altered in neurological conditions such as Alzheimer’s Disease and PAD [6]. Parkinson’s disease was associated with elevated TRP ratios, KYN, anthranilic acid (ANA), and kynurenic acid (KYNA), accompanied by reduced TRP concentrations, suggesting that enhanced peripheral production of kynurenine pathway metabolites may contribute to an increased risk of Parkinson’s disease [7]. Another study evaluating KP metabolites in L-DOPA-treated Parkinson’s disease patients found no significant differences in plasma KYN and 3-HK levels between the control and Parkinson’s disease groups [8].

Distinctive cytokine patterns mediate the periodontal inflammatory response by selectively recruiting inflammatory cells [9]. Patients with periodontitis are predisposed to diseases such as cardiovascular diseases, diabetes mellitus, and rheumatoid arthritis. There is a bidirectional relationship between PD and neurodegenerative diseases such as Parkinson’s and Alzheimer’s Diseases, where periodontitis is enhanced, and PD may exacerbate neurodegeneration [10].

Growing evidence suggests that systemic inflammation and immune dysregulation may represent a shared biological background linking Parkinson’s disease and periodontitis [10–14]. These processes may be mediated, at least in part, by activation of the tryptophan–kynurenine pathway through indoleamine-2,3-dioxygenase–driven inflammatory signaling, leading to the production of neuroactive and immunomodulatory metabolites that contribute to both neuroinflammatory degeneration and periodontal tissue destruction. We hypothesized that alterations in kynurenine pathway metabolism may reflect a shared biological mechanism linking Parkinson’s disease and periodontitis, contributing to compartment-specific neuroinflammatory and immunomodulatory alterations. Furthermore, we proposed that individuals with concomitant PAD and PD would exhibit distinct changes in TRP metabolism and downstream kynurenine metabolites in saliva and serum compared with subjects with either condition alone or healthy controls.

## Materials and methods

### Study population and ethical guidelines

Out of 326 patients who visited the Istanbul Medipol University Dental Hospital between 2022 and 2023, patients with Stage III, Grade B generalized periodontitis, as defined by the current classification scheme for periodontal and peri-implant diseases and conditions with and without Parkinson’s disease or periodontally and neurologically healthy, were recruited [15].

Each participant provided oral informed consent. The study was approved by the human subject ethics board of Istanbul Medipol University’s Faculty of Dentistry (date: 21/12/2023; Number: 1082) for the use and access of human subjects in research in accordance with the Helsinki Declaration of 1975, as revised in 2013. Parkinson’s disease was diagnosed using the United Kingdom Parkinson’s Disease Society Brain Bank criteria [16]. A.Z., a neurologist with experience in movement disorders, assessed each patient with Parkinson’s disease. The duration and pharmacologic management of PAD were documented. The PAD patients in this study underwent deep-brain stimulation therapy at least four months prior, and they were not diagnosed with Parkinson’s Plus Syndrome.

The general exclusion criteria included age younger than 18 years or older than 75 years; use of systemic antibiotics, anti-inflammatory or immunosuppressive medications, cardiovascular drugs influencing inflammatory responses, anticoagulants, or hormonal contraceptives within the previous three months; receipt of non-surgical periodontal therapy within the last six months or periodontal surgery within the past twelve months; presence of fewer than 20 natural teeth excluding third molars; and systemic or physiological conditions known to influence periodontal status, including diabetes mellitus, rheumatoid arthritis, pregnancy, lactation, alcoholism, smoking, or other relevant systemic diseases.

### Intra-examiner calibration

Prior to the study, the examiner (MY) was calibrated for clinical examinations. Ten volunteers were assessed twice, with a 1-hour interval between assessments, and the second set was performed blinded to the initial one. Calibration was accepted if the agreement between repeated clinical measurements was ≥ 90%.

### Clinical assessment of periodontal disease

The latest classification of periodontal and peri-implant diseases and conditions was used to diagnose the periodontal state based on previously established clinical and radiographic criteria. The clinical periodontal parameters of PI, PPD, CAL, and BOP were noted. All measurements were performed using a periodontal probe with William’s markings by a periodontist (MY).

### Saliva collection

Early morning hours were chosen for sample collection to minimize the impact of circadian rhythms on biomarker levels. Initially, distilled water was instructed to be used to rinse the mouth thoroughly. They were instructed to spit into the plastic tubes 5 times per minute for 10 min while sitting comfortably. The samples were then centrifuged at 2800×g for 10 min, the supernatant was carefully transferred into Eppendorf tubes, and the tubes were stored at -80 °C until analysis [17].

### Serum collection

Following saliva collection, the team member (NB) obtained blood samples. After centrifugation at 4000×g for 10 min, the serum was separated from the blood samples and stored at -80 °C until analysis [17, 18].

### Analysis of TRP, KYN, and KYN metabolites in saliva and serum samples

Tryptophan, kynurenine, and downstream kynurenine pathway metabolites (kynurenic acid, 3-hydroxykynurenine, picolinic acid, and quinolinic acid) were quantified using a modified liquid chromatography–tandem mass spectrometry (LC–MS/MS) protocol based on the method described by Tömösi et al. Briefly, 100 µL of saliva or serum was combined with 370 µL of an acetone–methanol solution containing 10 µL of an internal standard mixture. After vortex mixing for 60 s and incubation at − 20 °C for 15 min, the samples were centrifuged at 12,000 × g for 15 min at 4 °C. The resulting supernatant was transferred to a new tube, divided into two equal aliquots, and evaporated under a stream of nitrogen. Derivatization was performed by adding n-butanol–acetyl chloride (9:1, v/v), followed by incubation at 60 °C for 1 h. After a second nitrogen-evaporation step, the residues were reconstituted in 100 µL of the initial mobile phase. The aliquots were then recombined for the determination of kynurenine pathway metabolites. Assay linearity and precision were evaluated as previously reported [11].

### Statistical analyses

Sample size analysis was performed a priori using specific software^1^. Considering a large effect size (1) for the analysis involving three groups, an α-error of 0.05, and a power of 80%, the total sample size was 51 participants based on the KYN/TRP saliva ratio (0.452) reported by Onder et al. [11]. However, considering the possibility of confounders and incomplete data, the study was designed to include 59 patients. The normality of data distribution was determined using the Kolmogorov-Smirnov test. Differences between groups were determined using the analysis of variance (ANOVA) and Tukey post-hoc tests. The Kruskal–Wallis test and Bonferroni corrections were used for data that were not normally distributed. Data are presented as mean ± standard deviation or median. A General Linear Model (GLM) multivariate analysis was performed to control for age as a covariate. A value of *p* < 0.05 was considered significant.

## Results

### Study population and periodontal clinical parameters

Twenty patients with Parkinson+periodontitis [8 females, 12 males, and median (IQR) age: 58 (48.2–65.8) years], twenty-four systemically healthy patients with periodontitis [11 females, 13 males, and median(IQR) age: 51 (45.4–56.8) years)], and twenty-four systemically and periodontally healthy Control individuals [(C) group 13 females, 11 males and median (IQR) age: 40 (40-42.6) years)] completed all study procedures.

Demographic and periodontal clinical parameter data are presented in Table 1. Gender distribution showed no significant difference between the groups (*p* = 0.526). While age and clinical periodontal parameters (PPD, CAL, and BOP) were significantly higher in the Parkinson+periodontitis and periodontitis groups than in the control group (*p* < 0.001), they did not differ significantly between the two periodontitis groups. There were statistically significant differences among the age groups; however, a healthy age-matched control group could not be established. Therefore, GLM Multivariate Analysis was performed to control for age. No significant association was found between age and the KYN/TRP ratio (*p* = 0.548, partial eta-squared = 0.020). Both periodontitis groups (PD and PAD + PD) exhibited significantly higher PI values than the control group, with the periodontitis group presenting the highest statistically significant levels.

**Table 1:** Sociodemographic and periodontal clinical data

Age (years)	C (*n* = 24)	PD (*n* = 24)	PAD + PD (*n* = 20)	*p*
	40 (40-42.6)*	51 (45.4–56.8)	58 (48.2–65.8)	< 0,0001
Gender (F/M)	13/11	11/13	8/12	0,526
PD (mm)	1.31 ± 0.17*	3.48 ± 0.34	3.27 ± 0.21	**< 0**,**0001**
BOP (%)	3.54 ± 2.52*	70.46 ± 25.09	52.00 ± 14.91	**< 0**,**0001**
CAL (mm)	1.31 ± 0.17*	3.76 ± 0.53	3.42 ± 0.31	**< 0**,**0001**
PI	0.98 ± 0.34*	2.4 ± 0.36	1.91 ± 0.54**	**< 0**,**0001**

Control (C), Periodontitis (PD), Parkinson’s Disease+Periodontitis (PAD + PD), PPD: pocket probing depth, CAL: clinical attachment loss, PI: plaque index, BOP: Bleeding on probing. **Clinical periodontal parameters are presented as mean ± standard deviation (SD); ANOVA post hoc Tukey analysis. Age is presented as median (interquartile range**,** IQR); Kruskal-Wallis test with post hoc Bonferroni.** Gender is presented as frequency (n/n); Pearson chi-squared test. * C was statistically significantly different from PD and PAD + PD group (*p* < 0.001) ** Statistically significant difference between PD and PAD + PD (*p* < 0.05) Data are presented as mean ± standard deviation (SD)

According to the Hoehn and Yahr scale, 8 Parkinson’s disease patients (40%) were classified as stage 1, and 12 patients (60%) were classified as stage 2. All PAD patients were in the early stage of Parkinson’s disease (Hoehn and Yahr stages 1–2), corresponding to unilateral or bilateral involvement without postural instability and indicating mild to moderate clinical severity. The mean UPDRS Part III motor score for Parkinson’s disease patients was 19.15, indicating mild to moderate motor impairment (Table 2). The classification of medications used by PAD patients was as follows: Levodopa-piribedil-rasagiline (*n* = 3; PAD stage 2), Levodopa-rasagiline-pramipexole (*n* = 3; PAD stage 2), and amantadine-levodopa combination (*n* = 3; PAD stage 2). Additionally, the treatment regimens in stage 1 patients included piribedil-rasagiline (*n* = 2), rasagiline-levodopa combination (*n* = 2), apomorphine-levodopa combination (*n* = 3), and levodopa alone (*n* = 2).

**Table 2:** Distribution of disease stages and motor impairment severity in PAD patients

Parameter	Value
Hoehn and Yahr Stage 1	8 patients (40%)
Hoehn and Yahr Stage 2	12 patients (60%)
Mean UPDRS Part III Score	19.15

### Salivary and serum levels of TRP, KYN, KYN/TRP ratio, and KYN metabolites (KYNA, 3OHKYN, PA, QA) parameters

**Fig. 1:**
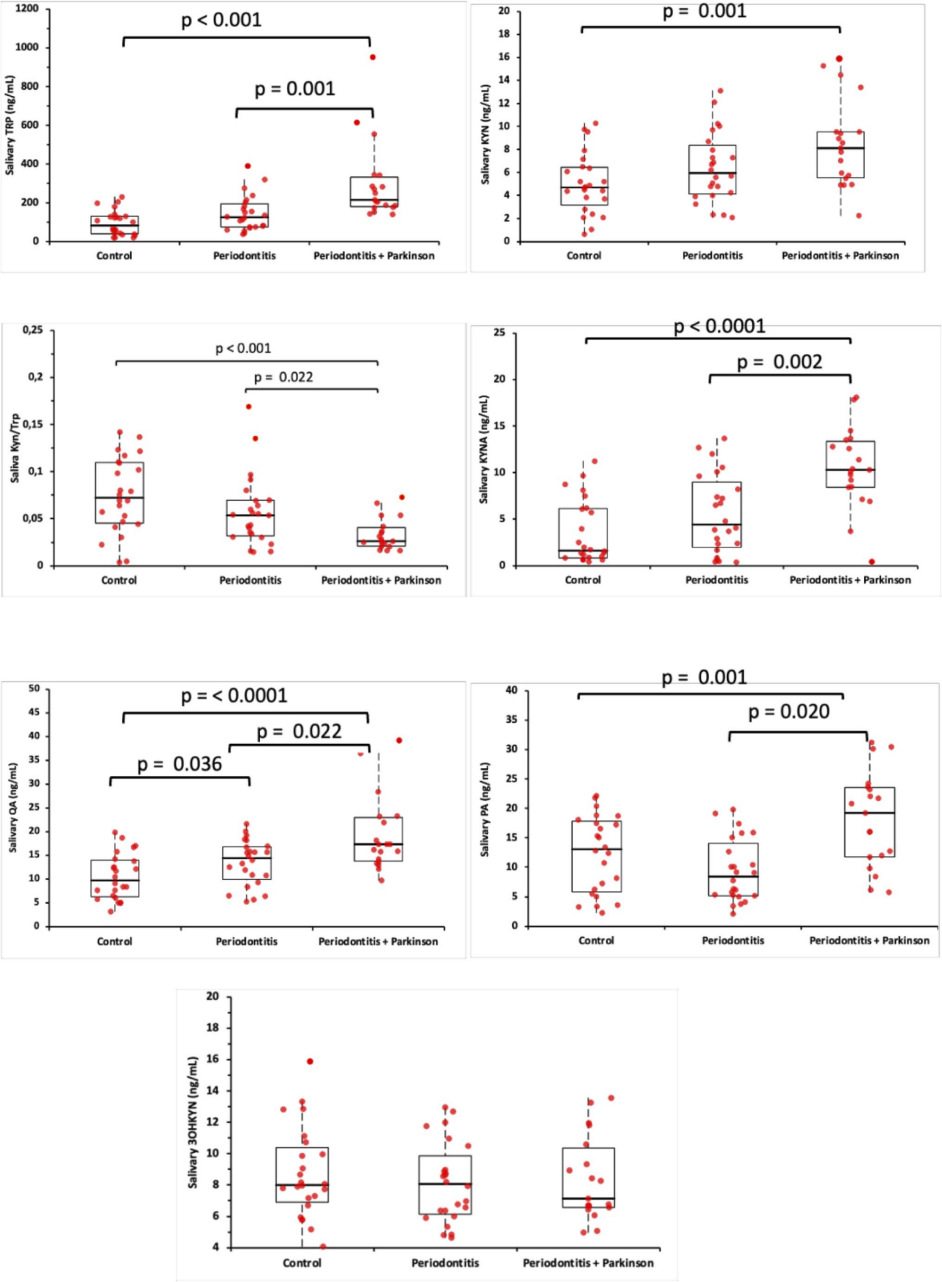
Boxplot showing salivary levels of TRP, KYN, KYN/TRP ratio, and KYN metabolites (KYNA, 3OHKYN, PA, QA) in patients and systemic healthy volunteers. 3OHKYN, 3 Hydroxykynurenine; KYN, Kynurenine; KYNA, Kynurenic Acid; PA, Picolinic acid; QA, Quinolinic acid; TRP, Tryptophan. The box represents the median and the 25–75% values; individual data points are overlaid as red circles. Kruskal-Wallis test posthoc Bonferroni. Statistical difference with the control group, *p* < 0.05

Salivary levels of TRP, KYN, KYN/TRP ratio, and KYN metabolites (KYNA, 3OHKYN, PA, QA) are presented in Fig. 1. In patients with Parkinson+periodontitis, salivary TRP, KYN, KYNA, PA, and QA levels were statistically significantly higher than in the healthy and periodontitis groups (*p* < 0.001 and *p* < 0.05, respectively). The KYN/TRP ratio was significantly lower (*p* < 0.001 and *p* = 0.022, respectively). QA is higher in the periodontitis group than in the control group (*p* < 0.001).

**Fig. 2:**
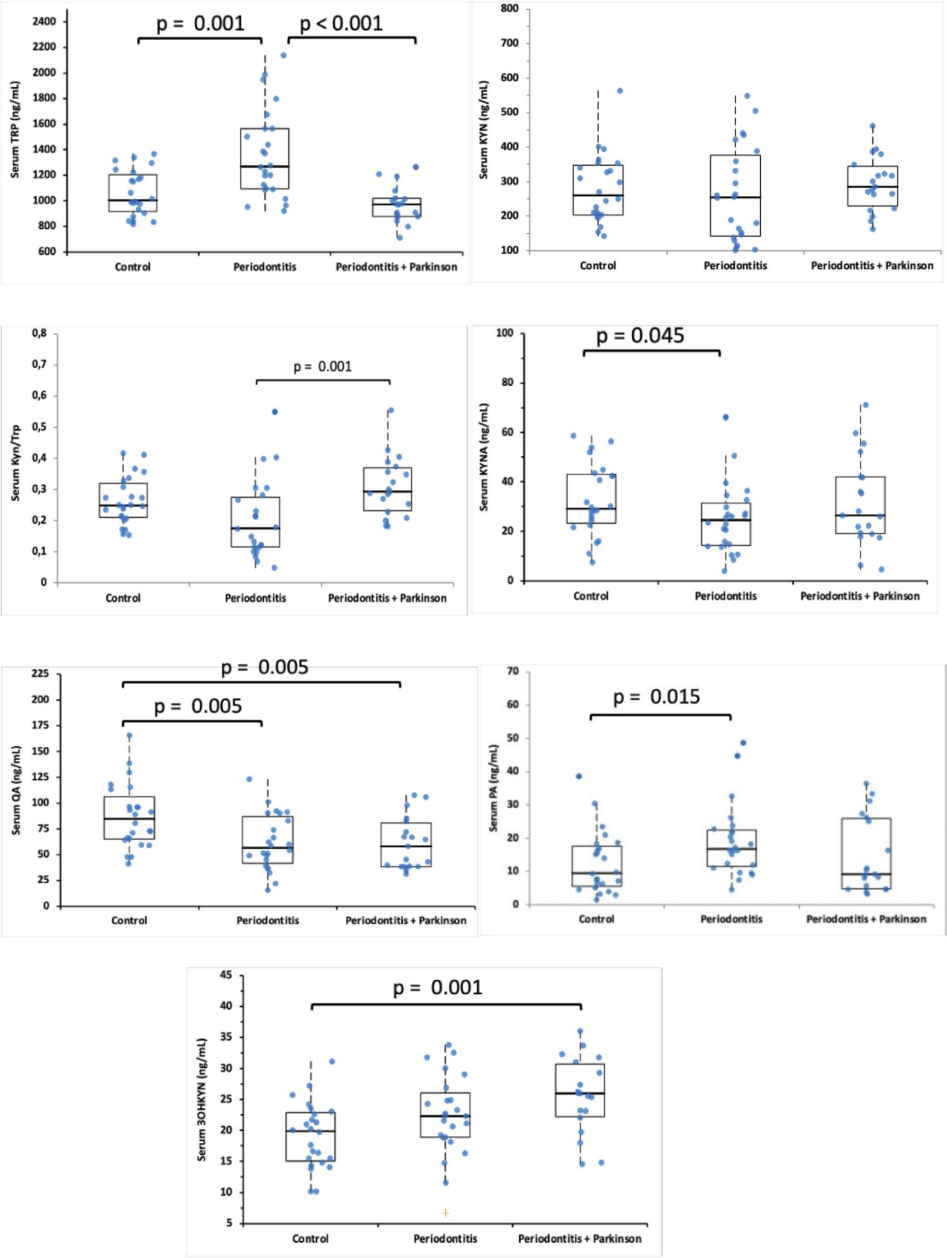
Boxplot showing serum levels of TRP, KYN, KYN/TRP ratio, and KYN metabolites (KYNA, 3OHKYN, PA, QA) in patients and systemic healthy volunteers. 3OHKYN, 3 Hydroxykynurenine; KYN, Kynurenine; KYNA, Kynurenic Acid; PA, Picolinic acid; QA, Quinolinic acid; TRP, Tryptophan. The box represents the median and the 25–75% values; individual data points are overlaid as blue circles. Kruskal-Wallis test posthoc Bonferroni. Statistical difference with the control group, *p* < 0.05

Serum levels of TRP, KYN, KYN/TRP ratio, and KYN metabolites (KYNA, 3OHKYN, PA, QA) are presented in Fig. 2. In the periodontitis group, serum TRP levels were significantly higher than in the controls and Parkinson+periodontitis group (*p* < 0.001). The serum KYN/TRP ratio is higher in the Parkinson+periodontitis group than in the periodontitis group (*p* < 0.001). Serum QA levels were higher in the control group than in both periodontitis and Parkinson+periodontitis groups (*p* = 0.005). Serum 3OHKYN levels were higher in the Parkinson+periodontitis group than in the control group (*p* = 0.001).

## Discussion

Parkinson’s disease affects a significant proportion of the global population, particularly in aging individuals, yet the underlying molecular and pathological mechanisms driving the disease remain incompletely understood [19, 20]. The kynurenine pathway contains both pro-inflammatory and neuroprotective components, which are two of the main pathways of tryptophan metabolism. Imbalances in this pathway are thought to accelerate the neurodegeneration process and exacerbate symptoms in Parkinson’s patients. Chronic inflammatory conditions such as periodontitis may influence Parkinson’s disease via systemic inflammatory mechanisms; however, this relationship is not conclusively established and should be interpreted cautiously. In this context, our study aimed to examine how these inflammatory pathways are reflected in patients with Parkinson’s disease and periodontitis [21–23].

In the presence of PD, the activation of the immune system triggered by pathogenic microorganisms leads to increased release of pro-inflammatory cytokines, initiating various biochemical pathways that drive the inflammatory response. This process leads to increased IDO enzyme expression and TRP catabolism, resulting in the accumulation of metabolic byproducts. A recent study reported that salivary TRP levels in individuals with periodontitis were higher compared to healthy participants, whereas no significant changes were observed in serum TRP levels [24]. In our study, salivary TRP levels in the periodontitis group alone did not differ significantly from those of the control group. When periodontitis was evaluated in the absence of Parkinson’s disease, the separation from healthy controls was limited, particularly in saliva, where only a single metabolite differed significantly. In contrast, several serum markers showed differences between periodontitis and control groups, suggesting that systemic measurements may be more sensitive to periodontal inflammation than salivary findings in this cohort. Overall, these results indicate that periodontitis alone may exert a modest influence on the kynurenine pathway, with different patterns observed in salivary and systemic measurements, whereas the coexistence of Parkinson’s disease appears to amplify these metabolic alterations. Notably, the salivary and serum findings did not fully parallel each other, particularly for TRP levels. While salivary TRP elevation was primarily observed in the Parkinson’s disease + periodontitis group, serum TRP levels were higher in the periodontitis group and did not differ significantly between controls and Parkinson’s disease + periodontitis participants. Rather than representing a contradiction, this divergence may reflect the distinct biological origins of salivary and systemic measurements. Salivary metabolites are strongly influenced by local periodontal inflammation, gingival crevicular fluid, and proteolysis driven by the oral microbiota, whereas serum TRP levels may be modulated by systemic host metabolism, neuroinflammatory activity, and dopaminergic therapy. Previous studies have also reported discordance between peripheral and local kynurenine-pathway markers in inflammatory and neurodegenerative conditions [25, 26]. Therefore, the present findings should be interpreted as indicating differential local and systemic regulation of tryptophan metabolism rather than a uniform systemic effect attributable to a single disease condition. As periodontal disease progresses, pathogenic bacteria can disrupt the protein content of the periodontium, leading to increased levels of various amino acids. Indeed, metabolomic analysis of saliva from patients with PD revealed enrichment of anaerobic proteolytic bacteria, such as *P. gingivalis*,* T. denticola*, and *T. forsythia*, in parallel with increased tryptophan metabolism [27]. In previous studies conducted by our team, we also showed that the subgingival microbiota composition in Parkinson’s patients with PD differed from that of patients with PD, particularly with an increased abundance of proteolytic bacterial species (e.g., *Aggregatibacter actinomycetemcomitans*,* Campylobacter rectus*,* Parvimonas micra*,* Treponema socranskii*,* T. denticola*,* Filifactor nodatum)* in the Parkinson’s group [28, 29]. Considering that salivary content is also derived from gingival crevicular fluid and, therefore, the subgingival microbiota, these findings suggested that the increase in proteolytic bacteria in the subgingival microbiota of the Parkinson+periodontitis group might be greater than that in the periodontitis group, leading to enhanced protein degradation and contributing to the increased salivary TRP levels observed in the Parkinson+periodontitis participants.

During periodontal disease, various cellular responses may slow TRP metabolism, leading to elevated blood levels. Two studies assessed serum TRP levels in patients with periodontitis and found no significant differences compared with healthy participants [11, 24]. In the treatment of PAD, various medications are utilized. Dopamine therapy can influence serotonergic pathways, promoting the conversion of tryptophan to serotonin, which may result in reduced serum TRP levels [8]. Considering that 16 participants in the Parkinson+periodontitis group were actively using L-dopa, the lower serum TRP levels observed in this group might be partially related to dopaminergic therapy rather than to disease-specific mechanisms.

Salivary KYN level in the Parkinson+periodontitis group was significantly higher than that of healthy participants. The activation of the TRP-KYN pathway in response to inflammation, resulting in increased KYN and its metabolites, is a predictable outcome. Although this increase did not reach statistical significance in the periodontitis group, Parkinson’s disease was associated with enhanced KYN production. In addition, the downstream metabolite KYNA also exhibited distinct variations in Parkinson’s disease. KYNA is produced through the transamination of KYN by kynurenine aminotransferases (KATs), primarily in the brain and peripheral tissues. As a neuroactive metabolite, KYNA plays a crucial role in modulating glutamatergic neurotransmission and protecting neurons from excitotoxicity, highlighting its potential relevance in the pathophysiology of Parkinson’s disease. A study in monkeys with Parkinson’s disease demonstrated that KYNA injection reduced motor symptoms [30]. KYNA in serum samples from patients with Parkinson’s disease is also increased^8^. In line with these studies, we observed that salivary KYNA levels in the Parkinson+periodontitis group were higher than those in the periodontitis and control groups. In this combined model, PD may alter tryptophan-kynurenine pathway activation by enhancing local inflammation, which could contribute to the increased salivary KYNA levels observed in patients with Parkinson+periodontitis. It has been reported that Parkinson’s patients exhibit reduced salivary secretion, which may also lead to higher KYNA concentrations in saliva. While KYN can be converted into KYNA via KAT enzymes, it can also undergo hydroxylation to produce 3-OHKYN, which possesses neurotoxic properties by generating free radicals and increasing oxidative stress [31]. Several studies have shown enhanced conversion of Kyn to 3-HK in brain regions associated with PA, leading to the subsequent generation of neurotoxic metabolites [32, 33]. A cohort study reported that serum 3-HK levels were higher in Parkinson’s disease participants compared to healthy individuals [26]. Similarly, in our study, serum 3-OHKYN levels in the Parkinson+periodontitis group were higher than those in the control group. However, because our study did not include a PAD-only group and the periodontitis group did not differ significantly from controls, this finding can only indicate that the coexistence of PAD and PD was associated with elevated systemic 3-OHKYN levels, rather than confirming the individual contribution of each condition.

Among the downstream metabolites of the tryptophan-kynurenine pathway, PA and QA share a common precursor, 2-aminomuconate semialdehyde. Their production is tightly regulated by metabolic conditions and inflammatory status, which in turn influence neuroprotection and neurotoxicity. PA exhibits metal-chelating and immunomodulatory properties, while QA is a well-established excitotoxic NMDA receptor agonist. Several studies indicated that PA exerted anti-inflammatory effects by suppressing TNF-α and NF-κB, regulating macrophage chemokines, inducing T cell immunosuppression, and mitigating oxidative stress and inflammation-related damage [34, 35]. In rabbits with induced PA, increased QA levels were observed in both brain and plasma samples [36]. Another study evaluating serum samples from Parkinson’s patients found no significant differences in QA and PA levels compared with healthy individuals. However, an increased QA/PA ratio was associated with greater difficulty performing daily functional activities [37]. According to our results, serum QA levels were lower, while PA concentrations were higher in the periodontitis and Parkinson+periodontitis groups compared to the control group. This finding could be interpreted as indicating that QA is rapidly metabolized systemically, thereby limiting its toxic effects. The inverse relationship between serum QA levels and PA suggested a potential metabolic shift toward PA and supported the notion that QA may be systemically regulated. The elevated salivary PA and QA levels observed in the Parkinson+periodontitis group compared to the periodontitis and control groups indicate an association between the coexistence of Parkinson’s disease and periodontitis and increased local inflammatory activity. However, since the study did not include a PAD-only group and the PD group did not differ significantly from controls, this observation cannot confirm whether the elevation in these metabolites results from the combined or individual effects of PAD and PD.

The KYN/TRP ratio is commonly used as an activation indicator, reflecting the activity of TDO and IDO enzymes. The KYN/TRP ratio tends to increase in diseases such as neurodegenerative disorders, Crohn’s disease, cancer, and depression [24, 38, 39]. Our KYN/TRP ratio results indicated an increased tryptophan catabolism in Parkinson’s patients, potentially linked to immune response and neuroinflammation.

This study has several limitations that should be considered when interpreting the findings. The cross-sectional design and relatively small sample size limit the ability to draw causal inferences. Additionally, age differences between groups and variations in anti-Parkinson therapy, particularly L-dopa use, may have influenced systemic tryptophan metabolism and biomarker levels. The absence of disease progression subgroups for both Parkinson’s disease and periodontitis limits the ability to evaluate stage-dependent metabolic alterations and reduces the generalizability of the findings across different severity levels. Given the preliminary nature of this investigation, the study scope was intentionally restricted to maintain clear, well-controlled analytical boundaries. Moreover, the lack of longitudinal follow-up restricts our understanding of whether these metabolic alterations precede or result from the coexistence of PAD and PD.

## Conclusions

Within the limitations of this cross-sectional study, individuals with coexisting Parkinson’s disease and periodontitis demonstrated distinct alterations in tryptophan–kynurenine pathway metabolites, characterized by divergent salivary and systemic patterns and a more pronounced metabolic shift in the combined disease state than in periodontitis alone. These findings support the concept of differential local and systemic regulation of tryptophan metabolism in neuroinflammation and periodontal inflammation, but do not establish causality. Further longitudinal and mechanistic studies are required to clarify the chronological and biological relationships underlying these observations.

## Data Availability

The research data generated and analyzed in this study derive from human clinical samples and are not publicly available due to privacy and ethical restrictions. However, anonymized data may be made available from the corresponding author upon reasonable request.

## Footnotes

1 3.0.10 G*Power; https://www.psychologie.hhu.de/arbeitsgruppen/allgemeine-psychologie-und-arbeitspsychologie/gpower.html.
